# Assessment of pain management adequacy among hospitalized pediatric patients: institutional-based cross-sectional study

**DOI:** 10.3389/fped.2023.1195416

**Published:** 2023-08-01

**Authors:** Asmamaw Emagn Kasahun, Ashenafi Kibret Sendekie, Rahel Belete Abebe

**Affiliations:** ^1^ Department of Pharmaceutics and Social Pharmacy, School of Pharmacy, College of Health Sciences, University of Gondar, Gondar, Ethiopia; ^2^ Department of Clinical Pharmacy, School of Pharmacy, College of Medicine and Health Sciences, University of Gondar, Gondar, Ethiopia

**Keywords:** pain, pediatric, analgesics, pain management prevention, pain management adequacy

## Abstract

**Background:**

As the evidence showed, despite the magnitude of the effects that pain can have on a child, it is often inadequately assessed and treated. However, whether pain is adequately treated or not, evidence is lacking in the study setting.

**Objectives:**

This study assessed pain management adequacy among hospitalized pediatric patients at the University of Gondar Comprehensive Specialized Hospital, Ethiopia.

**Methods:**

An institution-based cross-sectional study was conducted among pediatric patients admitted to the University of Gondar Comprehensive and Specialized Hospital between June and August 2021. Eligible patients were enrolled in the study using consecutive sampling techniques. Data were collected using a structured interview-based questionnaire and a review of the patient's medical records that were prepared after reviewing earlier studies. Pain management adequacy was determined using the pain management index (PMI) score. Statistical Software for Social Sciences (SPSS) version 22 was used for data entry and analysis. Descriptive statistics such as frequencies, percentages, and means with standard deviation were used to describe the respective variables. Logistic regression was used to assess predictor variables of pain management adequacy. A *p*-value <0.05 at a 95% CI was considered statistically significant.

**Results:**

Of the 422 participants enrolled in the study, most (58.1%) were males, with a mean age of 3.9 ± 0.8 years. Pain medication was prescribed to 62.8% (95% CI: 57.3–68.2) of the participants. About 63.3% (95% CI: 58.8%–68%) received inadequate analgesics. The type of painkillers administered also did not match the severity of the pain. Pediatric patients less than 1 month and between 1 month and 1 year (AOR = 2.891, 95% CI: 1.274–12.899 and AOR = 2.657, 95% CI: 1.350–5.175), respectively, and patients with severe and moderate levels of pain (AOR = 3.448, 95% CI: 1.902–6.251 and AOR = 5.345, 95% CI: 1.956–9.828), respectively, were found to have inadequate pain medication compared with their counterparts.

**Conclusion:**

This study revealed that pain was hardly managed based on its severity. Overall, two-thirds of pediatric patients received inadequate pain medication. This indicates majority of patients experienced pain did not manage appropriately.

## Introduction

Pain is an unpleasant emotion and sensory complaint associated with actual or potential tissue damage, as defined by the International Association for the Study of Pain (1–3). Pain is the “fifth vital sign,” and it should be assessed and recorded as usual with other vital signs (4–7). The appropriate intervention for pain is planned based on an accurate valuation of pain. Organized and routine pain assessment using standardized and validated measures is accepted as effective pain management in pediatric patients (8–10). Subjective experience and individual self-reporting are the favourite methods for assessing pain. Hence, when valid self-report is not available, as in children who cannot communicate due to age or developmental status, the observational and behavioural assessment tools are cornerstone substitutions (11).

Pediatric pain treatment protocols have made great progress recently with the advancement and validation of pain valuation tools for pediatric patients (12, 13). With all these advancements, the management of pain in pediatrics is still not well addressed. Unambiguously, neonates and infants are not managed for pain effectively, due to the misperception that they cannot sense pain as adults (14–16). Healthcare professionals’ who care for children are mainly responsible for eliminating or easing pain and suffering when possible (17, 18). The treatment of pain in childhood is like adult management practice, which includes pharmacological and non-pharmacological interventions (19). This management critically depends on an in-depth understanding of the developmental and environmental factors that influence nociceptive processing, pain perception, and the response to treatment during maturation from infancy to adolescence (20).

Data from children's hospitals around the world reveal that pain in pediatric patients from infancy to adolescence is common, under-recognized, and undertreated compared with adult patients (1–7, 21). Pediatric patients with the same diagnoses receive less pain management, and the younger the children are, the less likely it is that they receive adequate analgesia in the medical setting (22–25). Pediatric patients are the most undertreated and present to the hospital for pain compared to adults because of the wrong belief that they neither suffer pain nor remember painful experiences. Uncontrolled pain also has a direct impact on health outcomes and more than a few effects on all areas of life. The behavioral or emotional components of pediatric patients can also be used to assess pain and simplify the management practices (26). A long-term negative effect of untreated pain on pain sensitivity, immune functioning, neurophysiology, attitudes, and healthcare behavior is supported by numerous studies in pediatric patients. The lack of ability to notice pain, immaturity in remembering painful experiences, and other reasons make pain treatment more difficult in children than in adults (27).

A cross-sectional observational study in Toronto hospitals shows that out of the total 265 children, only 58.9% received a minimum of one documented intervention in pain management. Out of the 66 children with recognized pain, 55 received an intervention for their pain (20). The American Academy of Pediatrics (AAP) suggested that the lack of pain assessment and fears of adverse effects of pain medications, including respiratory depression and addiction, are the main barriers to the treatment of pain in children (18). As the underlying disease is expected to advance, a continuous adjustment of pain therapy is required. However, in 2013, in Jima, Ethiopia, a hospital-based study showed that only 44 of 162 children were prescribed pain medications, whereas two received a combination of acetaminophen and tramadol (28). To the extreme, only 11.1% of the patients received an intervention within the first 24 h of the onset of pain. The practice of assessing pain and its management in pediatric patients can show a discrepancy based on the different countries and their respective health institutions. As a result, efforts should be made to explore the inadequacy of pain management and prevention in this age group.

Generally, in consideration of the above challenges, management of pain in pediatric encompasses the use of pharmacological and non-pharmacological interventions to control the patient's identified pain. Studies on the adequacy of pain management and prevention among hospitalized paediatric patients are limited in resource-limited settings, particularly in Ethiopia. Therefore, this study assessed pain management and its adequacy in pediatric patients admitted to UoGCSH, Northwest Ethiopia. The study findings will add to the body of knowledge of both healthcare providers and patients and carers and will serve as the baseline for further study.

## Methods and materials

### Study design and setting

An institutional-based cross-sectional study was conducted among paediatric patients admitted to the University of Gondar Comprehensive and Specialised Hospital (UoGCSH) from June 17 to August 20, 2021. The hospital is among the largest teaching hospitals in the country and has provided both teaching and clinical services for different specializations, including pediatric. It serves more than five million people from its surroundings (29). This study was conducted on patients admitted to general wards paediatric wards (45 beds), malnutrition wards (25 beds), oncology wards (21 beds), and surgical paediatric (15 beds).

### Study participants and eligibility criteria

Pediatric patients less than 18 years of age who had experienced pain during their hospital stay in the UoGCSH were used as a source population. Pediatric admitted patients experienced pain during the data collection period were study participants and included in the study. Terminally ill patients, patients with neurological disorders, and individuals who refused to participate in the study were excluded.

### Sample size determination and sampling methods

The sample size was determined using the single population proportion formula with the assumption of a 5% margin of error, a 95% confidence interval, and a proportion *p* of 50%, as there were no previous studies found in the study area and a 10% non-response rate. The sample size was calculated as follows:

$$n=\frac{\left(Z\alpha /2)2p(1-p\right)}{d2}$$

where *n* is the minimum sample size required.

*Zα*/2 = the standard at the 95% confidence interval; *p* = proportion = 50%; *d* = margin of error, i.e., = 0.05.

$$n=\frac{\left(1.96)2(0.5)(1\text{–}0.5\right)}{(0.05)2}=384$$

With adding a possibility of 10% non-response rate, the final sample size was 422 hospitalized paediatric patients. Because of the limited number of patients admitted during the study period, participants were enrolled in the study by using the consecutive sampling techniques until an adequate sample size was reached.

### Operational definitions

The pain management index (PMI) was used to assess the adequacy of pain treatment in pediatric patients. PMI was calculated by subtracting the pain scores from the analgesics scores. The pain scores are classified as follows: 0 = no pain, 1 = mild pain, 2 = moderate pain, and 3 = severe pain. The analgesic scores derived from the WHO ladder are classified into the following: 0 = no analgesics, 1 = WHO I (non-opioid analgesics), 2 = WHO II (weak opioid), and 3 = WHO III (strong opioid). The Pain Management Index (PMI) ranges from −3 to 3; negative scores indicate undertreatment, but positive scores do not necessarily represent overtreatment.

Adequate pain management: it is indicated for the PMI score greater than or equal to zero, while inadequate pain management refers for the PMI score less than zero. Pain management: is an interdisciplinary approach for easing the suffering and improving the quality of life of those living with pain (30).

### Data collection instruments and procedures

A structured face-to-face interview-based questionnaire was developed for the participants interviews, while a semi-structured data extraction format was used for the collection of data from the participants’ medical records. The general data collection instrument was developed in English after an extensive literature review. It was translated to a local language, Amharic, to make the data collection procedure easy, then translated to English to maintain consistency. Different pain severity assessment tools were used to assess the level of pain. The revised Faces, Legs, Activity, Cry, and CONSOL Ability Scale (r-FLACC) tool was used to assess the severity of pain among pediatric who can't report pain by themselves (for those less than 5 years), while self-reporting pain assessment tools like the Faces Pain Scale-Revised tool (FPS- R Scale) and Numerical Pain Rating scales were used for participants who could report their pain by themselves, particularly, children aged greater than 5 years are capable of self-reporting their pain intensity (31, 32).

The data was collected by four graduating pharmacy students voluntarily. The participants’ interview method was employed using structured questionnaires for assessing pain and its management adequacy. Sociodemographic variables, diagnoses, comorbidities, and medications were collected variables. The prescribed analgesics, medication regimens, physician diagnosis, nursing care, and different pharmacological and non-pharmacological treatments were obtained from the patient's medical records. Before the data collection, both verbal and written consent was obtained from participants or carers. A parental consent form was signed for children less than or equal to 8 years old, while those aged above 8 years gave direct consent by themselves. Then, data collection procedures were conducted accordingly.

### Data quality control

Before the actual data collection period, the questionnaire was pretested on 5% of the study participants. A pretest was conducted at Debark General Hospital. Data collectors received a half-day training regarding the objectives, data collection producers, and ethical aspects. Then, data were collected after the enrolled participants were informed about the purpose of the study, the importance of their participation, and the ethical procedures. The collected data were checked for completeness, accuracy, and consistency at every step during and after data collection. The supervisors explicitly followed the data producers during the data collection period.

### Data entry, processing and analysis

Data was entered into SPSS version 22 for data processing and analysis. After the data was checked for its cleanliness, completeness, and consistency, analysis was carried out. The normal distribution of continuous variables was examined using a histogram, a Q-Q plot and the Shapiro-Wilk test. Categorical variables were presented using frequencies and percentages, while continuous variables were described by mean (standard deviation). Logistic regression was used to assess predictor variables of pain medications adequacy. Variables with a *p*-value <0.2 in the bivariable logistic regression were further analyzed in the multivariable logistic regression. The Hosmer-Lemeshow model was fitted and adjusted for variables. A *p*-value <0.05 at a 95% CI was considered as statistically significant.

## Results

### Enrollment and socio-demographic characteristics of the study participants

**Figure 1:**
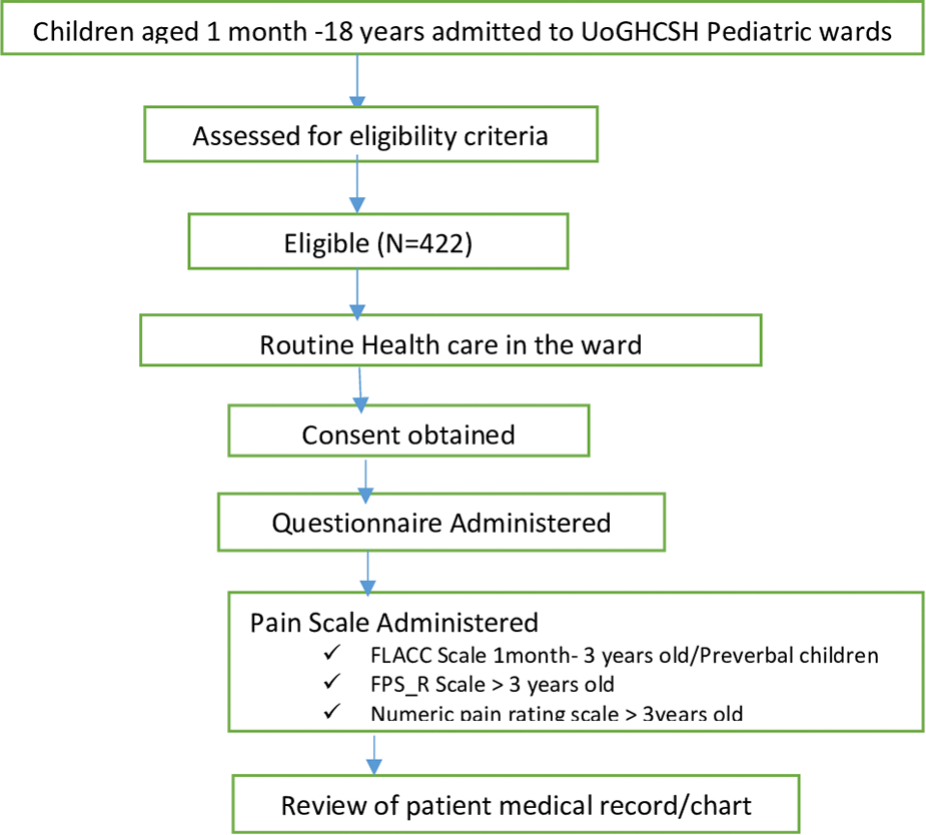
Flowchart detailing patient recruitment and data collection of pediatrics patients at UoGCSH (*N* = 422).

A total of 422 pediatric patients were enrolled in this study (100% response rate). The mean ± SD age of the study participants was 3.9 ± 0.8 years. Most of the study participants (58.1%) were males, with a higher proportion (37.4%) being toddlers or preschool age groups (1–5 years) (Table 1). The details of patient recruitment and data collection are shown in Figure 1.

**Table 1:** The sociodemographic characteristics of pediatric patients having pain at UoGCSH (*N* = 422).

Socio-demographics variables		Frequency (%)	Mean (±SD)
Sex	Male	245 (58.1)	
	Female	177 (41.9)	
Pediatrics age-based classifications	<1 month	28 (6.7)	3.9 (±0.8) years
	1 month–1 year	119 (28.2)	
	1 year–5 years	158 (37.4)	
	>5 years	117 (27.7)	
Weight (kg)	3	7 (1.7)	
	3–10	186 (44.1)	
	10–20	128 (30.3)	
	>20	101 (23.8)	
Height	<50 cm	23 (5.5)	68.7 (±8.9)
	50–100 cm	233 (55.2)	
	>100 cm	166 (39.3)	
Place of residence	Rural	173 (41.0)	
	Urban	249 (59.0)	
Health insurance usage	Yes	201 (47.6)	
	No	221 (52.4)	
Family monthly income (Eth. Birr)	1,500–2,999	171 (40.5)	4,650 (±235.6)
	3,000–4,999	145 (34.4)	
	>5,000	106 (25.1)	
Educational status of caregiver	Unable to read or write	146 (34.6)	
	Primary school	97 (23)	
	Secondary school	99 (23.5)	
	College and university	80 (19.0)	

### Patients’ pain description, severity and other clinical characteristics

**Figure 2:**
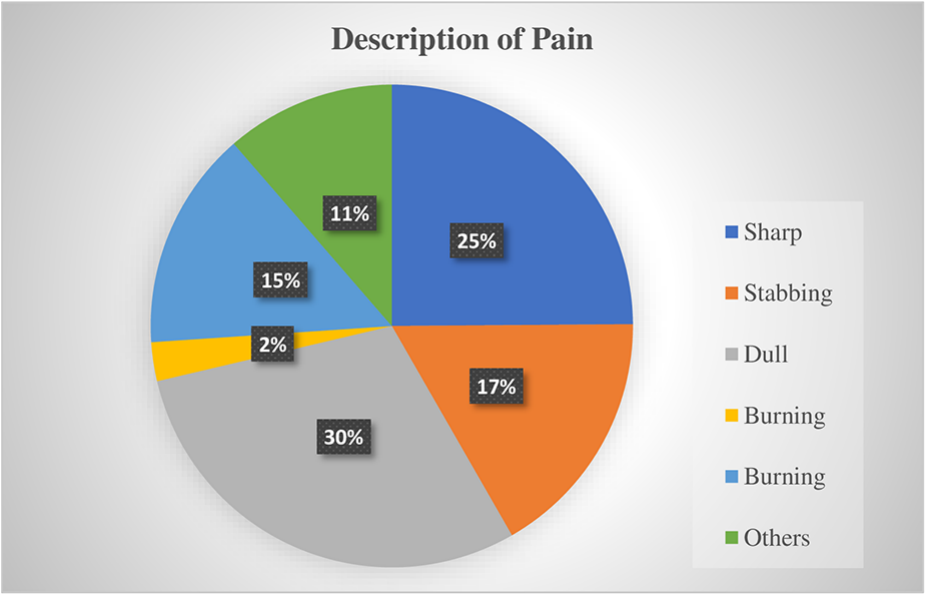
Patients’ pain description of pediatrics patients at UoGCSH (*N* = 422).

A higher proportion of patients (30%) experienced a dull type of pain. Detailed patient pain descriptions are illustrated in Figure 2. Regarding severity, moderate levels of pain were prevalent in a higher number of participants (38.4%). Around 35% of participants were interfering with daily activities because of pain. A significant number of participants were reported to have persistent pain (58.1%), and it frequently occurred (59.0%) (Table 2).

**Table 2:** Participants’ pain severity, description and other clinical characteristics of pediatrics patients at UoGCSH (*N* = 422).

Variables		Frequency (%)	Mean (±SD)
Severity (intensity)	Mild	143 (33.9)	
	Moderate	162 (38.4)	
	Severe	117 (27.7)	
Location	Upper extremity	86 (20.4)	
	Lower extremity	98 (23.2)	
	Abdomen	129 (30.6)	
	Respiratory system	56 (13.3)	
	Head	34 (8.1)	
	Others	19 (4.5)	
Duration from the onset of pain (days)	<3	127 (30.1)	4.2 (±0.7)
	3–6	194 (46.0)	
	6–10	74 (17.5)	
	>10	27 (6.4)	
Consistency of pain	Persistent	245 (58.1)	
	Breakthrough	177 (41.9)	
Frequency of pain	Rarely	173 (41.0)	
	Often	249 (59.0)	
	Usually	86 (20.4)	
	Always	16 (3.8)	
Pain interfering with daily activity	Yes	275 (65.2)	
	No	147 (34.8)	
Cause of pain (problems pain was related)	Abdominal problems	200 (47.4)	
	Musculoskeletal problems	131 (31.0)	
	Trauma	41 (9.7)	
	Others	50 (11.8)	
Physician diagnosis	Infection	101 (23.9)	
	Trauma	41 (9.7)	
	Surgical pain	25 (5.9)	
	More than one	131 (31.0)	
	Others	129 (30.6)	
Presence of previous pain experience	Yes	74 (17.5)	
	No	348 (82.5)	
Does the clinicians adequately assess your pain	Yes	179 (42.4)	
	No	243 (57.6)	
Length of hospital stay (days)	≤3	100 (23.7)	
	4–7	173 (41.0)	
	8–29	133 (31.5)	
	≥30	16 (3.8)	

### Participants’ pain medication patterns used in the management of pain

Less than two-thirds (62.8%) of the participants received pain medication. A higher proportion of the participants (39.6%) received a single acetaminophen analgesia. The most common routes of administration of the prescribed medications were oral (28.0%) followed by a suppository (20.6%). Most of the participants (58.8%) received pre-procedural pain medications. A higher proportion of the participants (30.1%) reported that they required an added dose or additional analgesics medication for the undertreated pain.

Beyond the pharmacologic interventions, participants were managed by non-pharmacological means, such as cold application (37.2%) followed by heat application (19.4%). The previously tried treatments for pain relief were prescription medications (15.6%) followed by OTC drugs (8.5%) and non-pharmacological (7.3%) (Table 3).

**Table 3:** Patterns of pain medications used in the pain management of pediatrics patients at UoGCSH (*N* = 422).

Variables		Frequency (%)
Pain medication received	Yes	265 (62.8)
	No	157 (37.2)
Number of pain medication received	One	222 (52.6)
	Two	36 (10.2)
	Three and above	7 (1.7)
Class of prescribed mediations	Acetaminophen	167 (39.6)
	NSAIDs	29 (6.9)
	Opioids	25 (5.9)
	Others^a^	4 (1.0)
Frequently prescribed/administered medications	Acetaminophen	167 (39.6)
	Diclofenac	18 (4.3)
	Ibuprofen	11 (2.6)
	Tramadol	12 (2.8)
	Morphine	13 (3.1)
	Others	4 (0.9)
Route of administration of prescribed medications	Oral	118 (28.0)
	Suppository	87 (20.6)
	Intravascular	23 (5.5)
	Intramuscular	7 (1.7)
	Others	5 (1.2)
Way of prescribed medication administration	PRN	123 (29.1)
	Scheduled based	120 (28.4)
	Non specified	179 (42.4)
Received pre-procedural pain medications	Yes	248 (58.8)
	No	174 (41.2)
Participants reported additional doses or added medications	Yes	127 (30.1)
	No	295 (69.9)
Adjuvant pain medication therapy	Glucocorticoids	21 (5.0)
	Anticonvulsants	21 (5.1)
	Bisphosphonates	9 (2.1)
Nonpharmacological management methods used	Cold	157 (37.2)
	Heat	82 (19.4)
	Massage	60 (14.2)
	Relaxation	31 (7.3)
	Distraction using pictures	7 (1.7)
	Changing positions	4 (0.9)
	Others	11 (2.6)
Participants perceive that they received adequate information about pain mediation treatment used	No	194 (46.0)
	Yes	228 (54.0)
Participants fear of the use of pain medications	Yes	77 (18.2)
	No	345 (81.8)
Qualification of the clinicians who prescribed pain medications	Specialist (Senior)	4 (1.0)
	Resident physician	68 (16.1)
	General practitioner	133 (31.5)
	Internship medicine students	198 (46.9)
	Bachelor's degree nurse	19 (4.5)
Previously used management to pain relief	The prescribed medications	66 (15.6)
	OTC Medications	36 (8.5)
	Non-pharmacological therapies	31 (7.3)
	Traditional/herbal management	12 (2.8)

^a^ Other steroids.

### Participants’ pain scores and management adequacy

**Figure 3:**
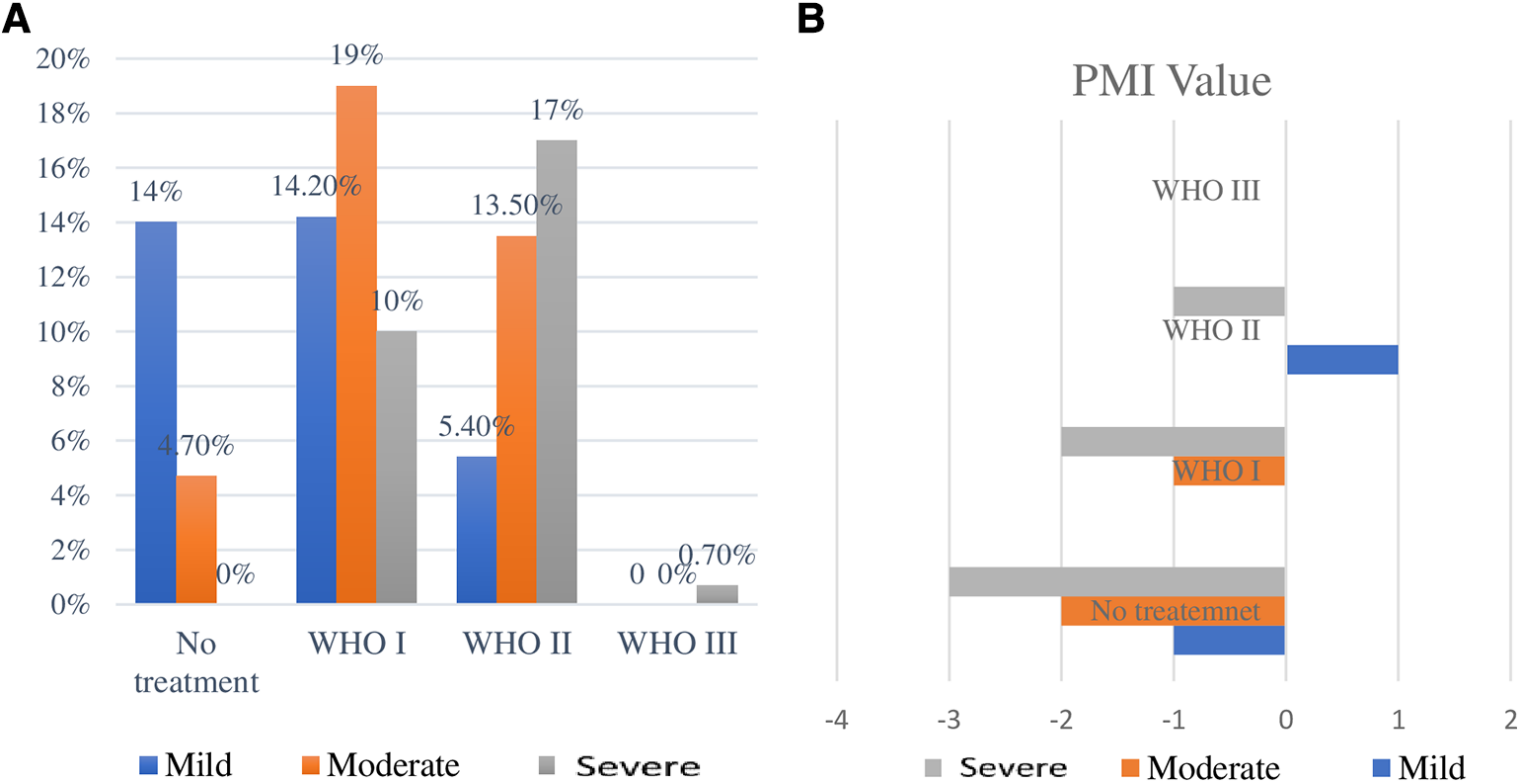
Proportion of participants with analgesics score at panel (**A**) and the pain medication they received based on WHO ladder classification and the value of the PMI at panel (**B**).

Although all children with severe pain received at least one pain medication, only a lower proportion (0.70%) of patients with severe pain received WHO III (strong opioid) management. Moreover, a higher proportion (4.7% and 14%) of patients with moderate and mild pain, respectively, did not receive any medication to relieve their pain (Figure 3A). The percentage of participants with negative PMI was about 63.3% (95% CI: 58.8%–68%). According to the index, those participants with a negative PMI were being inadequately treated. The value of PMI was positive only in a lower proportion of children with mild pain who received weak opioid analgesics (WHO II), +1, which is illustrated in Figure 3B. The intensity of pain and PMI of the study subjects were expressed in Figure 3.

### Determinants of pain management adequacy

Overall, only 36.7% (95% CI: 32%–41.2%) of the pediatric patients received adequate analgesia. The association of pain management adequacy and potential predictor variables was assessed using a logistic regression model. As a result, a multivariable logistic regression analysis showed that the age of patients and intensity of pain have a significant association with pain treatment adequacy. After taking other variables constant, pediatrics patients aged less than 1 month and those from 1 month to 1 year were found more likely to have inadequate pain management compared with those aged greater than 5 years (AOR = 2.891, 95% CI: 1.274–12.899 and AOR = 2.657, 95% CI: 1.350–5.175), respectively. Similarly, patients with severe and moderate levels of pain were found to be more likely to have inadequate pain management than those with mild levels of pain intensity (AOR = 3.448, 95% CI: 1.902–6.251 and AOR = 5.345, 95% CI: 1.956–9.828), respectively (Table 4).

**Table 4:** Association of pain management adequacy with predictor variables of pediatrics patients at UoGCSH (*N* = 422).

Variables		Pain management adequacy		95% CI		*p*-value
		Inadequate	Adequate	COR	AOR	
Sex	Male	149	96	0.776 (0.518–1.163)	0.482 (0.290–1.799)	0.085
	Female	118	59	1	1	
Age	<1 month	16	12	1.224 (0.534–14.211)	2.891 (1.274–12.899)	0.004*
	1 month–1 year	102	37	2.531 (1.501–4.268)	2.657 (1.350–5.175)	0.004*
	1 year–5 years	98	60	1.499 (0.924–2.434)	2.617 (0.650–3.074)	0.073
	>5 years	61	56	1	1	
Residence	Rural	85	88	0.356 (0.236–0.535)	0.762 (0.139–2.054)	0.061
	Urban	182	67	1	1	
Educational status of participants/care givers	Unable to read and write	84	62	0.339 (0.179–0.641)	0.920 (0.386–2.191)	0.371
	Primary school	59	38	0.388 (0.196–0.768)	0.619 (0.266–1.441)	0.451
	Secondary school	60	39	0.385 (0.195–0,759)	0.959 (0.419–2.192)	0.782
	College and university	64	16	1	1	
Health insurance usage	Yes	121	80	0.777 (0.523–1.155)	0.793 (0.170–3.886)	0.113
	No	146	75	1	1	
Monthly income family (Eth. birr)	1,500–2,999	103	68	0.994 (0.606–1.631)	1.093 (0.586–2.038)	0.253
	3,000–4,999	100	45	1.458 (0.863–2.464)	1.552 (0.831–2.899)	0.278
	≥5,000	64	42	1	1	
Intensity (severity) of pain	Severe	74	43	2.753 (1.662–4.561)	3.448 (1.902–6.251)	<0.001*
	Moderate	138	24	9.200 (5.313–15.930)	5.345 (1.956–9.828)	<0.001*
	Mild	55	88	1	1	

CI, confidence interval; COR, crude odds ratio; AOR, adjusted odds ratio.

* Indicates *p* < 0.05.

## Discussion

Based on the authors’ best searching strategy, no single study that assessed pain management in the paediatric population in the study setting has been published yet. The current finding is fundamental to highlighting the extent of pain management adequacy. This hospital-based cross-sectional study assessed pain management adequacy among admitted paediatric patients. The findings from this study highlighted the management pattern and whether the pain is adequately treated or not based on its WHO class of severity. Additionally, the findings explored potential predictor variables associated with pain management inadequacy. The study revealed that about two-thirds of the patients received inadequate pain medication. Paediatric patients younger than 1 year and patients with severe and moderate levels of pain, respectively, were found to have inadequate pain medication compared with patients older than 5 years and patients with mild pain.

Pain is among a common symptoms in hospitalized children (33). Assessment of pain and optimal treatment in pediatric patients is critical, as inadequate acute pain management can cause chronic pain (34, 35), which lead to morbidities like posttraumatic stress symptoms (36). Additionally, inadequately managed pain can result in adverse physical and psychological patient consequences at the patient level, families and communities at large. Pain should be assessed and managed at the initial stages. Assessing the cause of pain might also be important. In this study, the most frequent cause of pain was related to abdominal problems. Thus, pain management, considering the nature of the cause and individualization, could be important. However, adequate management of pain needs more knowledge of children's self-rating of pain intensity experience (37). To enhance pain management and communication between healthcare providers in health facilities, pain and treatment adequacy should be routinely monitored and documented (38). Evidence also showed that the way to proper pain management is to implement validated pain assessment tools and to invest in education (39, 40). Despite adequate pain management in pediatric patients is of extreme importance, few studies have demonstrated.

In this study, the prevalence of moderate to severe pain was high among hospitalized children, and its management was suboptimal. This is consistent with previous studies (2, 3, 21, 41–43). This finding may indicate that pediatric patients are not received pain medications based on their level of pain severity properly. This might be related to a lower assessment practice of pain in pediatric patients. Therefore, assessing pain using a pain intensity rating tool is an important part of pain management to identify the intensity of pain and to indicate and evaluate pain management (31, 32, 44).

In the management of pain, a multimodal approach with two or more medications has been recommended in pediatric patients with persistent underlined pain (45), as well as for those with acute and post-operative pain (31–33). In contradiction to the evidence, a higher proportion of the patients in this study received a single acetaminophen-based analgesia than the majority of pain medications. However, the evidence indicates that a combination of pain medications that target different mechanisms in different systems, either in the central or peripheral nervous system have a more effective pain relief effect compared with a single analgesic medication (31).

In line with the previous studies (31, 32, 44, 45), the current study showed that pediatric patients received different modalities of non-pharmacological management modalities. Physical therapies (cold or hot therapy), psychological support, distractions, relaxation, and massage were among the common treatments used in admitted pediatric patients. The findings imply that although pharmacological treatment is part of a comprehensive approach towards the treatment of pain, nonpharmacological methods play an undeniable role in relieving pain in pediatric patients. They play a prominent role in the management of chronic pain, in particular. Pictures, music, and computer games are among the non-pharmacological approaches of pain management that can actively distract children's attention away from the pain and procedures (46).

The other interesting aspect that this study demonstrated is the association between pain management adequacy and predictor variables of inadequate pain management. Although the educational background of caregivers, health insurance status, and monthly household income had an association with pain management adequacy in bivariable regression, only the age of the patients and severity of pain were found to have a significant association with pain management adequacy with further analysis using multivariable logistic regression. Consequently, the study found that pediatric patients aged less than 1 year were more likely to have pian management inadequacy compared with those patients with an age greater than 3 years. This might be related to a lower pain assessment in infants because of their difficulty self-reporting their symptoms and pains. Children of a lower age, particularly infants, couldn't report their pains comprehensively. As a result, they sustain the inadequacy of management unless there is a comprehensive pain assessment and management practice. The findings may imply that pediatrics patients, particularly infants, might require special attention towards assessing and managing their pains. Therefore, clinicians could be motivated in the manner of pain management practices. They should adhere to standard tools and guidelines while managing pediatric patients, in particular infant patients.

Consistent with previous studies (21, 41, 47), the current study showed that patients with severe and moderate levels of pain were found to be more likely to receive inadequate pain medications compared with those with a mild level of pain. These studies revealed that severe pain is more prevalent and that patients are more likely to experience inadequate pain management. Unrelieved pain, in particular severe pain, has harmful effects on patients. It could be visualized that patients suffer from pain in many ways. This could be critical for the lives of patients. Patients with pain become depressed, and it is psychologically unsafe to have pain.

Generally, the current study highlighted the adequacy of pain management and the extent of pediatric patient pain treatment in a resource-limited setting. Furthermore, it also demonstrated the need to explore the factors associated with pain medications inadequacy. Therefore, we also hope to give some insight into and add to the body of knowledge for practitioners and patients related to the adequacy of pain treatment and prevention in pediatric patients.

### Strength and limitations of the study

This study is the first of its kind to assess the adequacy of pain management in paediatric patients and can be a fundamental tool to highlight the extent of pain management. As a limitation, pain was not assessed under different conditions, such as at rest, during movement, and during sleeping time. Because of the nature of the study design, we took the data at the appointed time, but we could not assess the response to pain management and were unable to follow undertreated patients. Point prevalence does not adequately describe the quality of pain management in a population because of these reasons. The PMI is also limited by assessing adequacy based on type of analgesics for severity of pain rather than dose, which results in a very crude measure of treatment adequacy according to the WHO guidelines. The PMI also does not distinguish adherence to the guidelines or the quality of the prescribers. A mixed study method, including a qualitative study, might be important and welcomed for future research. Despite these limitations, this study can provide insight regarding the extent of pain management practice among pediatric patients in a resource-limited setting.

## Conclusion

This study concluded that the majority of pediatric patients received inadequate pain management. Patients’ age and level of severity of pain were found to be significantly associated with pain management inadequacy. Pain management in pediatric patients could include compliance with guidelines, focusing on the severity of pain, and age-based assessment and management. Since pain is linked to different systems and has multifactorial causes, a combination of pain medications can provide a better outcome to relieve the pediatric patients from persistent pain. Therefore, patients could be assessed according to their severity, and adequate management should be tailored accordingly. Clinicians could also be highly vigilant and motivated towards adherence to pain assessment and management practices in pediatric patients. Additionally, nurses who care for pediatric patients could become familiar with the assessment tools and management modalities.

## Data Availability

The raw data supporting the conclusions of this article will be made available by the authors, without undue reservation.
